# An Unusual Case of Metastatic Basal Cell Carcinoma of the Prostate: A Case Report and Literature Review

**DOI:** 10.3389/fonc.2020.00859

**Published:** 2020-05-27

**Authors:** Shiqiang Dong, Qing Liu, Zihan Xu, Haitao Wang

**Affiliations:** Department of Oncology, The Second Hospital of Tianjin Medical University, Tianjin Institute of Urology, Tianjin, China

**Keywords:** basal cell carcinoma, prostate, metastasis, case report, therapy

## Abstract

**Background:** Primary basal cell carcinoma (BCC) is a rare prostate cancer. Currently, a standard treatment regime for BCC of the prostate is lacking and most patients have a poor prognosis. We reported on a patient with BCC of the prostate whose cancer metastasized after undergoing a radical prostatectomy and whose prognosis improved after treatment with etoposide.

**Case Presentation:** A 62-year-old male with a history of seminoma was admitted complaining of intermittent gross hematuria for 1 month. Following a prostate biopsy, the patient was diagnosed with BCC of the prostate and received radical prostatectomy and radiotherapy. Initially, the patient's symptoms improved; however, 2 years later, a chest computed tomography (CT) scan revealed lung nodules. The patient did not exhibit any symptoms of BCC of the prostate; however, pathological examination and immunohistochemical staining of the nodules confirmed metastatic BCC of the prostate. Chemotherapy with docetaxel and cisplatin was well-tolerated but did not slow disease progression. Next-generation sequencing revealed mutations in the ataxia telangiectasia-mutated (*ATM*), SWI/SNF-related matrix-associated actin-dependent regulator of chromatin subfamily b-member 1 (*SMARCB1)*, and phosphoinositide-3-kinase regulatory subunit 1 (*PIK3R1*) genes. The patient did not receive targeted therapy owing to financial limitations and instead, etoposide was administered. A 9-month follow-up chest CT scan showed an 80% reduction in existing lung nodules and no new nodules had developed.

**Conclusion:** Our patient, diagnosed with recurrent prostate BCC after receiving a radical prostatectomy, responded to treatment with etoposide. Radical prostatectomy and radiotherapy should remain first-line therapy; however, etoposide may be an alternative second-line therapy when other options are not available. Consensus regarding treatment plans, and the molecular mechanisms behind prostate BBC, must be elucidated.

## Introduction

Basal cell carcinoma (BCC) is most frequently observed in areas of the body that receive sun exposure, including the skin, and BBC of the prostate is extremely rare. Until recently, only 99 cases of primary BCC of the prostate had been reported (1). Typically, BCC of the prostate possesses low malignancy potential; however, there are reports of aggressive BBC leading to metastasis and recurrence (2–5). Owing to the limited number of cases, proper management strategies are still lacking for prostate BCC. We reported on a 62-year-old male who showed a partial response to etoposide, according to the Response Evaluation Criteria in Solid Tumors 1.1 (RECIST 1.1).

## Case Description

Approximately 2.5 years ago, a 62-year-old male who presented with intermittent gross hematuria and a prostate-specific antigen (PSA) level of 2.42 ng/mL was admitted to the Tianjin Baodi hospital. A digital rectal examination did not reveal any masses or nodules. Ultrasound examination revealed an enlarged prostate (3.4 × 4.6 × 3.3 cm) and the bladder is normal. He did not present any psychosocial disorders and no one in his family had been diagnosed with a tumor. A 12-core prostate biopsy revealed BCC in one-half of the prostate. A pathology report was obtained from the hospital where the patient was initially diagnosed. The prostate biopsy was immunohistochemically negative for PSA, alpha-methyl acyl-coenzyme A racemase, chromogranin A, and synaptophysin; and positive for cytokeratin-903 (34βE12), p63, and Ki67 (<1%). An abdominal computed tomography (CT) scan was normal indicating there had not been metastasis. This was confirmed by whole-body bone scintigraphy. Based on these findings, the patient was diagnosed with non-metastatic prostate BCC and was treated with a radical prostatectomy. The pathology report indicated local invasion of the nerve and thrombosis of tumor vessels; however, the margin and seminal vesicles were negative. Immunohistochemical analysis was negative for PSA, alpha-methyl acyl-coenzyme A racemase, P53, cytokine 7 (CK7), and CK20 and positive for 34βE12, p63, and Ki67 (<1%).

**Figure 1:**
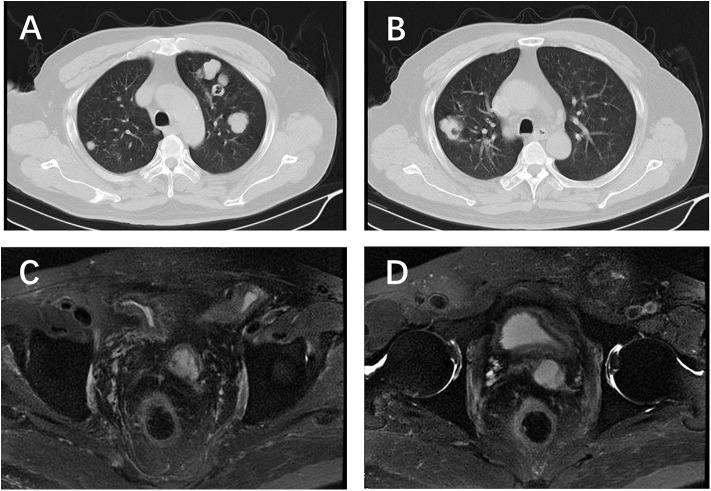
Chest CT and pelvic MRI before etoposide chemotherapy. **(A,B)** Multiple nodules located in both sides of the lung; **(C,D)** no visible recurrence shown on the pelvic MRI.

**Figure 2:**
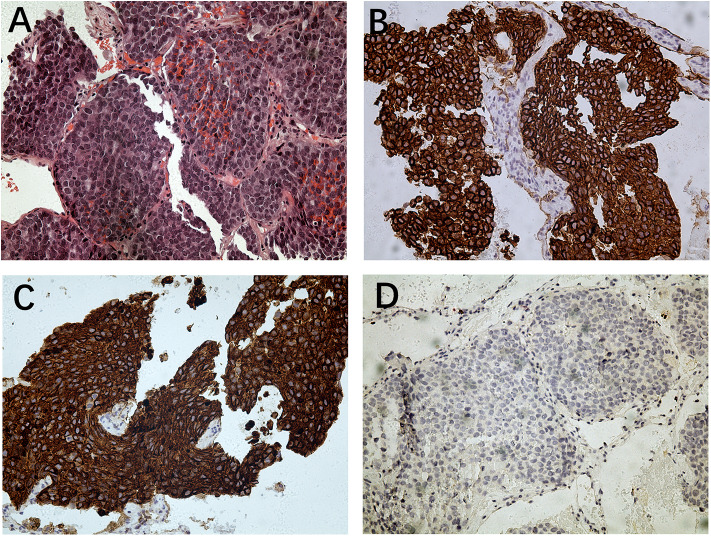
Histopathology of lung metastesis. **(A)** Hematoxylin and eosin staining (magnification: ×200); **(B)** immunohistochemistry for 34βE12 (magnification: ×200); **(C)** immunohistochemistry for CK5/6 (magnification: ×200); **(D)** immunohistochemistry for PSA (magnification: ×200).

The patient's TNM classification was pT2NxMx. To reduce the risk of metastasis, our patient received image-guided radiotherapy. The serum PSA level remained unchanged at 0.00 ng/mL before and after radiotherapy. The patient showed no evidence of disease progression until he was medically examined 2 years later. A chest CT scan revealed multiple lung nodules (Figure 1); however, bone scintigraphy showed no metastasis. A biopsy was performed on the nodules, and the patient was diagnosed with metastatic BCC of the prostate. Immunohistochemical analysis was negative for PSA, chromogranin A, synaptophysin, androgen receptor, thyroid transcription factor-1, CD117, CD30, and octamer-binding transcription factor-4 and positive for 34βE12, P40, CK5/6, low molecular weight cytokeratin, and Ki67 (20%) (Figure 2).

**Figure 3:**
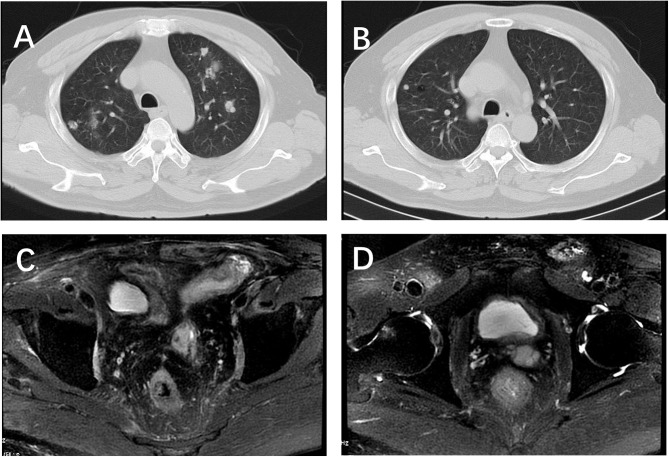
Chest CT and pelvic MRI after 5 circles etoposide chemotherapy. **(A,B)** 80% decrease in size of measurable lung nodules; **(C,D)** no visible recurrence shown on the pelvic MRI.

Information regarding the management and outcomes for BCC of the prostate is limited, and there is currently no standard treatment. We reviewed relevant literature to determine the optimal diagnostic and treatment methodology. Hormonal therapy is commonly administered; however, outcomes are poor. Considering that basal cells do not exhibit secretory activity, the patient received three cycles of chemotherapy with docetaxel, but the tumor continued to grow, albeit slowly. Subsequently, we added cisplatin for another three cycles of chemotherapy. This treatment failed and the number of nodules in the patient's lungs increased. Therefore, next-generation sequencing of the patient's sample from a lung nodule was performed free of cost at Foundation Medicine, which revealed mutations in the ataxia telangiectasia-mutated (*ATM*), SWI/SNF-related matrix-associated actin-dependent regulator of chromatin subfamily b-member 1 (*SMARCB1*), and phosphoinositide-3-kinase regulatory subunit 1 (*PIK3R1*) genes. The patient did not receive targeted therapy owing to his financial limitations and there were no clinical trials in which he could enroll. The patient's Zubrod/ECOG/WHO score was 1; therefore, following approval by the ethics committee, the patient received nine cycles of chemotherapy with etoposide (100 mg/day for 10 days, 4 weeks/cycle) after disease recurrence to prolong survival. A 9-month follow-up chest CT scan revealed a nearly 80% reduction in the size of lung nodules (Figure 3).

## Discussion

Owing to the rarity of BCC of the prostate, when this type of prostate cancer is detected, physical examinations, abdominal CT scans, and magnetic resonance imaging should be performed to exclude the possibility of metastasis. The 2016 WHO classification of tumors of the urinary system and male genital organs categorizes adenoid cystic hyperplasia carcinoma and basaloid variants as malignant basal cell tumors. A basaloid pattern is characterized by irregular solid clumps, trabeculae, and large cellular masses of basaloid cells. Tumor cells contain small, dark, often angulated nuclei, and a scant cytoplasm forming small nests in a peripheral palisading pattern (4). Infiltrative permeation, extraprostatic extension, perineural invasion, necrosis, and stromal desmoplasia are characteristic of BCC and these characteristics may assist in differential diagnosis. Immunohistochemical analyses revealed that most BCC cells were positive for B-cell lymphoma-2, 34βE12, p63, and CK5/6 (6). Previous studies reported mutations in the MYB proto-oncogene (*MYB*), phosphatase and tensin homolog (*PTEN*), epidermal growth factor receptor (*EGFR*), and erb-b2 receptor tyrosine kinase 2 (HER-2) genes (7–10). Simper et al. demonstrated that PTEN expression is downregulated and EGFR is overexpressed in BCC cells (8). Of the 99 cases of prostate BBC reported (mean age: 67.1 ± 12.2 years), clinical data were available for only 88 cases. Of these 88 patients, 33 (37.5%) patients whose cancer had not metastasized underwent radical prostatectomies, including 3 receiving pelvic exenterations; metastasis occurred in 17 patients and was undetermined in 52, whereas cancer in 19 patients remained localized. Eight of the 17 patients showed metastasis after surgery and 9 showed metastasis when diagnosed. Locations of metastases included the liver, lung, bone, penis, colon, and seminal vesicles. The liver (64.7%) was the most common area for metastasis. The percentages of lung and bone metastasis were 58.8 and 35.3%, respectively. Of the 71 patients for which follow-up data were available, 15 (21.1%) lived ≤1 year and only 20 (28.2%) lived ≥5 years.

When next-generation sequencing was performed, mutations in *ATM, SMARCB1*, and *PIK3R1* were revealed. ATM is a serine/threonine-protein kinase that plays a critical role in DNA damage responses. Mutations in *ATM* can lead to a defective DNA damage response and homologous recombination-mediated DNA repair. *ATM* mutations induce sensitization to PARP inhibitors such as olaparib, the only PARP inhibitor approved by the National Medical Products Administration of China for epithelial ovarian cancer, dermal ovarian cancer, fallopian tube cancer, and primary peritoneal cancer (11–13). Loss or inactivation of ATM may increase the sensitivity to PARP inhibitors or inhibitors of DNA-dependent protein kinase subunit (14). Sun et al. demonstrated that inhibition of the ATM pathway can increase p53 activation, apoptosis, and accumulation of DNA damage (15). *SMARCB1* encodes the SNF5 protein (also known as INI1), which is one of the three core subunits of the SWI/SNF family of chromatin remodeling complexes (16). Preclinical evidence suggests that the loss of *SMARCB1* can increase the sensitivity to the enhancer of zeste 2 polycomb repressive complex 2 subunit inhibitors (17), inhibitors of the Hedgehog pathway (18), CDK4/6 inhibitors (18), and inhibitors of the fibroblast growth factor receptor (19). *PIK3R1* encodes the p85-alpha regulatory subunit of phosphatidylinositol 3-kinase (PI3K) (20). The loss of PIK3R1 can result in increased PI3K signaling and promote tumorigenesis and hyperplasia in PTEN-deficient cells (21). Preclinical studies have shown that mutations in *PIK3R1* may increase sensitivity to the PI3K-AKT-mTOR pathway inhibitors, specifically inhibitors of PI3K-alpha or AKT. Moreover, PIK3R1 plays an important role in conferring resistance to cisplatin (22). Drugs targeting SMARCB1 and PIK3R1 are still being evaluated in clinical studies.

Therapeutic treatment options for patients with BCC of the prostate are limited because of the rarity of this disease. Most patients with primary BCC of the prostate are treated with hormone therapy, radiotherapy, radical prostatectomy, or a combination of these treatments. However, outcomes remain poor. Our patient initially received a radical prostatectomy and radiotherapy. His progression-free survival over a 17-month period between initial treatment and reoccurrence was monitored. Six cycles of chemotherapy with docetaxel and cisplatin did not reduce the number or size of the lung nodules. Following this treatment failure, nine cycles of chemotherapy with etoposide were administered, producing a partial response resulting in an 80% decrease in the size of existing lung nodules and no development of new nodules. During etoposide treatment, the patient showed only mild nausea and vomiting. Thus, although some studies have shown that etoposide is ineffective in patients with BCC of the prostate (6, 23); in certain case like the present one, etoposide may be the most suitable option. The mechanism of action of etoposide in patients with prostate BCC should be further studied. A CT scan of the patient with BCC of the prostate revealed his condition was stable 9 months after commencing treatment with etoposide.

## Ethics Statement

Written informed consent was obtained from the individual(s) for the publication of any potentially identifiable images or data included in this article.

## Author Contributions

SD: manuscript writing. QL: data collection. ZX: data collection. HW: project development and data collection.

## Conflict of Interest

The authors declare that the research was conducted in the absence of any commercial or financial relationships that could be construed as a potential conflict of interest.

## Abbreviations

BBC: basal cell carcinoma
*ATM*: ataxia telangiectasia-mutated
*SMARCB1*: SWI/SNF-related matrix-associated actin-dependent regulator of chromatin subfamily b-member 1
*PIK3R1*: phosphoinositide-3-kinase regulatory subunit 1
PSA: prostate-specific antigen
*PTEN*: phosphatase and tensin homolog
*EGFR*: epidermal growth factor receptor.
